# Outcomes of sustained fetal tachyarrhythmias after transplacental treatment

**DOI:** 10.1016/j.hroo.2021.02.006

**Published:** 2021-03-09

**Authors:** Raphael Bartin, Alice Maltret, Muriel Nicloux, Yves Ville, Damien Bonnet, Julien Stirnemann

**Affiliations:** ∗ Obstetric and Maternal Fetal Medicine and EA7328; † M3C-Necker, Pediatric and Congenital Cardiology Unit; ‡ Neonatology and Neonatal Intensive Care Unit; § Hôpital universitaire Necker-Enfants malades, AP-HP; ‖ Université de Paris, Paris, France

**Keywords:** Congenital heart defect, Echocardiography, Fetal arrhythmia, Fetal ultrasound, In utero therapy, Tachyarrhythmia, Ultrasound

## Abstract

**Background:**

Fetal tachyarrhythmia is a condition that may lead to cardiac dysfunction, hydrops, and death. Despite a transplacental treatment, failure to obtain or maintain sinus rhythm may occur.

**Objective:**

We aimed to analyze the perinatal outcomes of sustained fetal tachyarrhythmias after in utero treatment.

**Methods:**

We performed a retrospective evaluation of 69 cases with sustained fetal tachyarrhythmia. We compared the perinatal and long-term outcomes of prenatally converted and drug-resistant fetuses. Tachyarrhythmia subtypes were also evaluated.

**Results:**

Conversion to sinus rhythm was obtained in 74% of cases; 26% of cases were drug-resistant and delivered arrhythmic. Three perinatal deaths occurred in both groups (6.7% vs 17%, *P* = .34). Neonates delivered arrhythmic were more frequently admitted to neonatal intensive care units (75% vs 31%, *P* < .01), and their hospital stay was longer (20.9 vs 6.64 days, *P* < .001). Multiple neonatal recurrences (81% vs 11%, *P* < .001), temporary hemodynamic dysfunction or heart failure (50% vs 6.7%, *P* < .001), and postnatal use of a combination treatment (44% vs 13%, *P* = .028) were also more frequent in this population. Beyond the neonatal period, rates of recurrences within the first 16 months were higher in drug-resistant fetuses (HR = 16.14, CI 95% [4.485; 193.8], *P* < .001). In this population, postnatal electrocardiogram revealed an overrepresentation of rare mechanisms, especially permanent junctional reciprocating tachycardia (PJRT) (31%).

**Conclusion:**

Prenatal conversion to stable sinus rhythm is a major determinant of perinatal and long-term outcomes in fetal tachyarrhythmias. The underlying electrophysiological mechanisms have a major role in predicting these differential outcomes with an overrepresentation of PJRT in the drug-resistant population.

**Key Findings**

▪ The diagnosis of electrophysiological mechanisms in fetal tachyarrhythmias is difficult and remains uncertain during the prenatal period in a significant proportion of cases.
• Prenatal conversion to sinus rhythm is associated with significantly better outcomes.
▪ In drug-resistant cases, rare mechanisms such as permanent junctional reciprocating tachycardia or atrial ectopic tachycardia are overrepresented, for which the perinatal management is challenging.
▪ Although restoring sinus rhythm significantly reduces postnatal morbidity, adverse perinatal events and recurrences occur. Perinatal and long-term monitoring should be performed at specialized centers, even following successful prenatal conversion to sinus rhythm.

## Introduction

The prevalence of fetal arrhythmias appears to be around 1%–2% of pregnancies, although it is probably underestimated, as intermittent arrhythmias and spontaneous resolution may occur.^1^^,^^2^ The most common type of arrhythmia is ectopic atrial beats, a benign condition in 95% of cases.^3^ However, fetal tachyarrhythmia, mostly supraventricular tachycardia (SVT), is a potentially severe condition that may lead to fetal hydrops, heart failure, and intrauterine fetal death.^2^^,^^4^ The causes of SVT have been well established with 2 main mechanisms: atrioventricular reciprocating tachycardia (AVRT) through an accessory pathway (AP) and atrial flutter, accounting for 70%–80% and 20%–30% of fetal tachyarrhythmias, respectively.^5^ Other causes of SVT, such as permanent junctional reciprocating tachycardia (PJRT), junctional ectopic tachycardia, or atrial ectopic tachycardia (AET), are less frequent.^2^^,^^6^^,^^7^

The rationale for transplacental antiarrhythmic treatment (TPT) is to restore sinus rhythm to prevent hydrops and intrauterine death, and potentially allow for vaginal delivery when stable sinus rhythm has been obtained.^8^^,^^9^ Although the overall efficacy of medical transplacental therapies is undisputed, failures and recurrences occur,^10^, ^11^, ^12^, ^13^ accounting for a large proportion of the perinatal morbidity and mortality of the condition. The objective of this study is to analyze the perinatal outcomes of fetal tachyarrhythmias after in utero treatment.

## Methods

### Study population

All consecutive cases of sustained tachyarrhythmias referred to our department for perinatal management following the diagnosis of fetal tachyarrhythmia between January 2008 and August 2019 were reviewed. Tachyarrhythmia was diagnosed when heart rate was ≥180 beats per minute (bpm) and was considered sustained if present during ≥50% of the echocardiographic monitoring time^2^^,^^14^; otherwise, it was considered intermittent. We excluded fetuses with congenital heart disease and genetic or chromosomal associations.

All cases were assessed by echocardiography. M-mode and aortic pulsed Doppler were used to sequentially analyze atrial and ventricular electrical activity. Whenever possible, the underlying mechanism of prenatal tachyarrhythmia was specified. Atrial tachycardias were diagnosed when the atrial electrical activity was faster than the ventricle’s. In case of atrial flutter, atrial activity usually ranges from 350 to 500 bpm with a 2:1 or 3:1 AV conduction leading to a slower ventricular frequency (200–250 bpm). AET, owing to ectopic atrial activity, may display a 1:1 or variable AV conduction. In case of reciprocating tachycardia, a short interval between the ventricular and atrial activity is in favor of a typical AP (short V-A interval), with sudden onset and offset. PJRT displays a long V-A interval owing to reentry by a decremental AP. No junctional ectopic tachycardia was observed in this cohort.

### Prenatal treatment

All cases of sustained tachyarrhythmias were analyzed, including fetuses referred after failure of a first attempt to prenatal cardioversion. Therefore, we did not evaluate the efficacy of our TPT protocol, which was not standardized. The number of treatment lines, which was defined by a change in medication, or the introduction of a new medication in combination with the previous one was retrieved from the patient’s files. Echocardiography was performed every 2 days to assess heart rhythm and tolerance. A new treatment line was initiated in the absence of conversion after 5 days of treatment, or in case of worsening hydrops or cardiac dysfunction. If initiated in our department, TPT was generally started within 24 hours following echocardiographic assessment. Digoxin was the main first-line treatment especially given to fetuses without hydrops. The dose is adapted until maternal serum level reaches therapeutic ranges (1–2 ng/mL), which were regularly monitored. In case of hydrops, flecainide was usually the preferred first-line treatment, alone or associated with digoxin. Flecainide was also used as a second line after failure of digoxin alone. Treatment was started at 300 mg per day, only before 36 weeks. Serum levels were not measured. Amiodarone was considered only for second- or third-line treatment, given the potential adverse effects on maternal and fetal thyroid, while monitoring the maternal thyroid function. Beta-blockers, such as sotalol or propranolol, are also considered for second-line treatment.

Prenatal invasive therapies such as fetal direct administration of antiarrhythmic drugs (ie, intracordal or intraperitoneal) or in utero transesophageal pacing^15^ were considered in cases of sustained tachyarrhythmia despite multiple treatment lines with worsening hydrops and cardiac failure.

For drug-resistant fetuses, delivery was discussed based on gestational age, evolution of hydrops or fluid effusions, cardiac function, and treatments tried. If the risk of prematurity was considered lower than trying a new line of TPT, cesarean section was performed.

Postnatal recurrences or drug-resistant cases were treated using either amiodarone, digoxin, or propranolol. Amiodarone was initiated with a loading dose of 500 mg/m^2^ during 5–7 days and then lowered at 250 mg/m^2^, with thyroid function follow-up. Digoxin was initiated between 5 and 10 μg/kg (half if associated to amiodarone). Propranolol was given at 3 mg/kg/day.

Given the risk of postnatal recurrence of atrioventricular reentrant tachyarrhythmia, initiation of a prophylactic treatment after birth was left to the discretion of each cardiologist. When initiated, treatment was maintained for 6–12 months, according to European guidelines.^16^ Patients with reduced atrial flutter were not treated. Postnatal follow-up included regular clinical examinations, echocardiography, and Holter electrocardiogram (ECG).

### Outcomes

Fetal hydrops was characterized by the presence of the following findings: ascites, pleural or pericardial effusions, or skin edema. The severity of hydrops was graded as follows: moderate hydrops when only 1 serosa effusion was observed; severe hydrops when >1 effusions or skin edema was found.

Perinatal death is defined as in utero fetal deaths or postnatal death occurring <28 days. Tachyarrhythmia was considered converted when the fetus showed stable sinus rhythm throughout follow-up, with initial postnatal ECG confirming sinus rhythm (group 1). Drug-resistant tachyarrhythmias were defined by persistent arrhythmia at birth, confirmed by neonatal ECG (group 2). Combination therapy was defined as the use of 2 or more different treatments (including beta-blockers, digoxin, amiodarone) to obtain stable sinus rhythm after birth. Neonatal hemodynamic dysfunction was defined as clinical and echocardiographic signs of heart failure (including cardiac arrest), or the need for inotropic or vasoactive drugs in addition to restoration of sinus rhythm.

Postnatal recurrences are defined as recurrences occurring in an infant following prenatal conversion to sinus rhythm with an ECG confirming sinus rhythm at birth and were separated into 2 categories:

(1) Neonatal recurrences, which referred to tachyarrhythmia events occurring during the neonatal period, immediately after delivery and prior to 28 days of life. Multiple neonatal recurrences were defined by at least 2 episodes of tachyarrhythmia requiring treatment modification (add-on antiarrhythmic therapy or drug change).

(2) Long-term recurrences, which were defined as recurrences beyond the neonatal period, with a follow-up starting after hospital discharge and up to 16 months. All recurrences were confirmed by Holter ECG.

### Statistical analysis

All statistical analyses were conducted using R (http://www.r-project.org; R Foundation, Vienna, Austria). The Mann-Whitney test was used to compare differences in continuous variables between 2 groups. Categorical data was analyzed using Fisher exact test. Long-term recurrence-free survival rates at 16 months were estimated using Kaplan-Meier curves. Data were censored at the time of last visit. Comparison between drug-resistant and prenatally converted fetuses was assessed by log-rank test and hazard ratio (HR) using a Cox model. Statistical significance was defined by a *P* value < .05.

### Ethical statement

Institutional Review Board approval was waived owing to the use of retrospective and de-identified data. The research reported in this paper was conducted according to the principles of the Declaration of Helsinki. All mothers provided their written consent for data use.

## Results

Seventy-three fetuses were diagnosed with sustained tachyarrhythmia between January 2008 and August 2019. Four cases were excluded: 1 associated congenital heart defect; 1 associated severe cerebral lesions and 1 long-QT syndrome for which the parents opted to terminate the pregnancy; 1 lost to follow-up before delivery.

**Figure 1:**
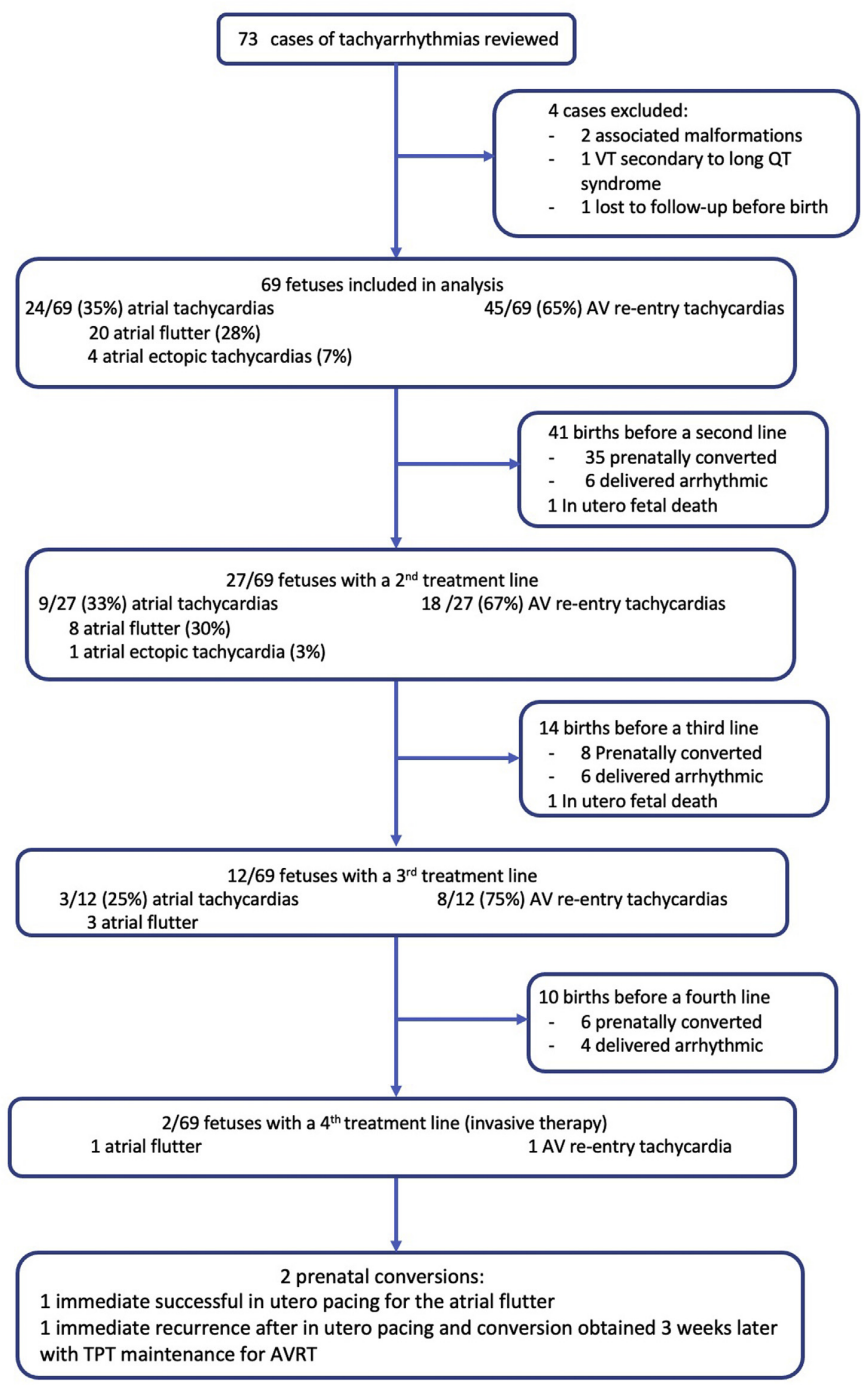
Flow chart of the population with different lines of transplacental treatment / fetal interventions. AVRT = atrioventricular reciprocating tachycardia; transplacental antiarrhythmic treatment; VT = ventricular tachycardia.

Figure 1 shows the flow chart of fetuses with the number of TPT lines, rates of success to obtain sinus rhythm, and rhythmic status at birth. Postnatal data were incomplete in 6 cases and were excluded for the analysis of postnatal outcomes.

### Prenatal outcomes

TPT was successful to obtain stable sinus rhythm in 51 of 69 fetuses (group 1). No intrauterine fetal death occurred in group 1. TPT failed in 18 of 69 fetuses (24%) (group 2). Two of the 18 fetuses died in utero in group 2. The remaining 16 fetuses were delivered arrhythmic. Table 1 compares fetal characteristics and pregnancy management between prenatally converted in sinus rhythm cases and drug-resistant cases. In both groups, the majority of fetuses had atrioventricular reentrant tachycardias (63% and 72%, respectively). Expectedly, the presence of hydrops and preterm birth were significantly associated with drug-resistant arrhythmia.

**Table 1:** Population characteristics, management, and delivery outcome according to prenatal conversion of tachyarrhythmia to sinus rhythm or failure to obtain sinus rhythm

		Prenatal conversion to sinus rhythm Group 1	Drug-resistant Group 2	*P*
		N = 51	N = 18	
Fetal echocardiographic diagnosis, n (%)	AV re-entry	32 (63%)	13(72%)	.25
	AFl	17 (33%)	3 (17%)	-
	AET	2 (3.9%)	2 (11%)	-
Heart rate (beats/min)		230 [210; 259]	230 [210;250]	.96
GA at diagnosis (weeks)		30.4 [25.0; 33.1]	31.6 [29.0;33.8]	.22
Prenatal hydrops, n (%)	None	32 (63%)	6 (33%)	.029
	Moderate	10 (20%)	3 (17%)	-
	Severe	9 (18%)	9 (50%)	-
Treatment lines, n (%)	0–1	34 (67%)	7 (39%)	.12
	2	9 (18%)	7 (39%)	
	3–4	8 (15%)	4 (22%)	
Amiodarone, n (%)		10 (20%)	6 (33%)	.33
GA at birth (weeks)		38.7 [37.6; 39.2]	36.7 [34.4; 37.7]	<.01
Birth weight (g)		3255 [2860; 3585]	3120 [2700; 3330]	.14
Cesarean section, n (%)		18 (40%)	15 (94%)	<.001
IUD, n (%)		0 (0%)	2 (11%)	.067
Perinatal death, n (%)		3 (6%)	3 (17%)	.34

AET = atrial ectopic tachyarrhythmia; AFl = atrial flutter; GA = gestational age; IUD = intrauterine demise; PJRT = permanent junctional reciprocating tachycardia; SVT = supraventricular tachyarrhythmia.

Two cases were treated by fetal transesophageal pacing. One was a case with a severe hydropic fetus diagnosed with atrial flutter at 27 5/7 weeks of gestation, which showed worsening hydrops despite 2 TPT lines. Fetoscopy was performed at 29 4/7 weeks of gestation and cardioversion was achieved without further recurrence. This case was published previously.^15^ A second case presenting with drug-resistant AVRT complicated with hydrops at 23 4/7 weeks of gestation received intraperitoneal injection of digoxin and in utero pacing after 2 weeks and 3 TPT lines. Despite a successful attempt, tachyarrhythmia recurred rapidly after the procedure. However, conversion to sinus rhythm was finally obtained 1 month later with TPT.

### Neonatal outcomes

Neonatal outcomes are presented in Table 2. Compared to prenatally converted fetuses, drug-resistant fetuses were more frequently admitted to the neonatal intensive care unit (NICU) (75% vs 31%, *P* < .01), and their hospital stay was longer (20.9 vs 6.64 days, *P* < .001). Multiple neonatal recurrences (81% vs 11%, *P* < .001) and temporary hemodynamic dysfunction or heart failure (50% vs 6.7%, *P* < .001) were more frequent in drug-resistant tachyarrhythmias. The need for combination of antiarrhythmic drugs was required more frequently in group 2 (44% vs 13%, *P* = .028), as antiarrhythmic treatment was mainly prophylactic in group 1 patients.

**Table 2:** Postnatal management and outcome according to success or failure of prenatal conversion to sinus rhythm

	Prenatal conversion to sinus rhythm Group 1	Drug-resistant Group 2	*P*
	N = 45	N = 16	
Hospitalization (days)	5.00 [3.00; 7.00]	16.0 [11.8; 18.5]	<.001
Admission to NICU, n (%)	14 (31%)	12 (75%)	<.01
Multiple neonatal recurrences, n (%)	5 (11%)	13 (81%)	<.001
Combination treatment, n (%)	6 (13%)	7 (44%)	.028
Hemodynamic dysfunction, n (%)	36 (38%) (6.7%)	8 (50%)	<.001
Postnatal hydrops, n (%)			<.01
- None	42 (93%)	9 (56%)	
- Moderate	1 (2.2%)	1 (6.2%)	
- Severe	2 (4.4%)	6 (38%)	

NICU = neonatal intensive care unit.

**Figure 2:**
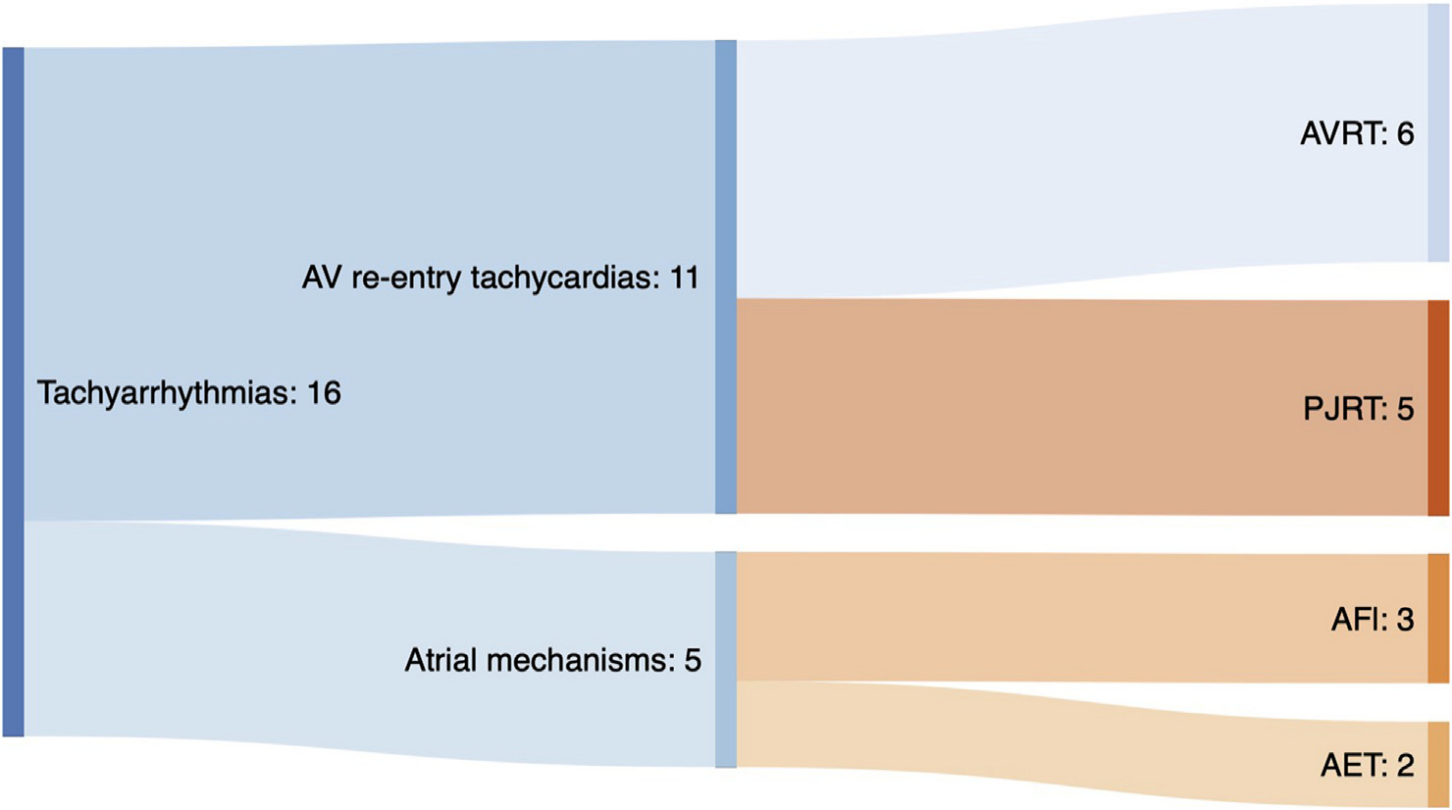
Postnatal assessment of tachyarrhythmia mechanisms of drug-resistant fetuses. AET = atrial ectopic tachyarrhythmia; AFl = atrial flutter; AVRT = atrioventricular reciprocating tachycardia; PJRT = permanent junctional reciprocating tachycardia; SVT = supraventricular tachyarrhythmia.

Figure 2 shows the final mechanisms of SVT in fetuses born alive and arrhythmic assessed by ECG, and Holter ECG or transesophageal ECG when needed. Of the 16 neonates born alive with drug-resistant tachyarrhythmia, 11 cases had an AV reentrant tachyarrhythmia (6 AVRT, 5 PJRT) and 5 had an atrial tachyarrhythmia (3 atrial flutter, 2 AET).

Six of the 45 neonates born in sinus rhythm had neonatal recurrences and 5 of the 6 had multiple neonatal recurrences. Admission to NICU, hemodynamic dysfunction, and need for antiarrhythmic combination therapy were similar in these patients compared to those born arrhythmic. Two of these neonates died postnatally (details below). The 4 other cases with neonatal recurrence were delivered at term with good neonatal tolerance and favorable outcome. The mechanism of tachyarrhythmia in these neonates was AVRT in 4 cases and dual mechanism (atrial flutter and AVRT) in the remaining 2 cases.

### Overall mortality

Three perinatal deaths occurred in both groups

In group 1, no in utero death was observed. In 1 case, the fetus (with initial diagnosis of atrial flutter with severe hydrops) was converted to sinus rhythm after 3 lines of TPT, but the mother developed severe pre-eclampsia (mirror syndrome), and cesarean section was performed at 31 4/7 weeks. The child presented a recurrence on a dual mechanism (atrial flutter and AVRT) and died of necrotizing colitis. In a second case, emergency cesarean section was performed at 29 1/7 weeks of gestation for decreased heart rate variability on cardiotocography. No specific cause was found and the neonate died of necrotizing colitis. The third patient initially had AVRT with severe hydrops. While converted by flecainide only, hydrops persisted, and severe bradycardia was observed on a follow-up ultrasound. Despite immediate delivery at 33 3/7 weeks, the neonate died of multiorgan failure in the NICU.

In group 2, 2 fetuses died in utero. These cases had severe hydrops and intrauterine death was considered directly caused by the tachyarrhythmia. One fetus presented severe bradycardia in the context of severe pre-eclampsia, with placental insufficiency (permanent reverse flow in the umbilical artery). Severe hypoxic-ischemic encephalopathy was diagnosed, and the neonate died in the NICU. The difference in the proportion of perinatal death between the 2 groups was not found to be statistically significant (6.7% vs 17%, *P* = .34).

### Long-term follow-up

Long-term follow-up was available for 57 surviving children after first hospital discharge. Two of them had a follow-up shorter than 16 months: 1 in the drug-resistant group, who presented a recurrence at 2 months of age; and 1 prenatally converted to sinus rhythm, who remained in sinus rhythm without recurrence.

**Figure 3:**
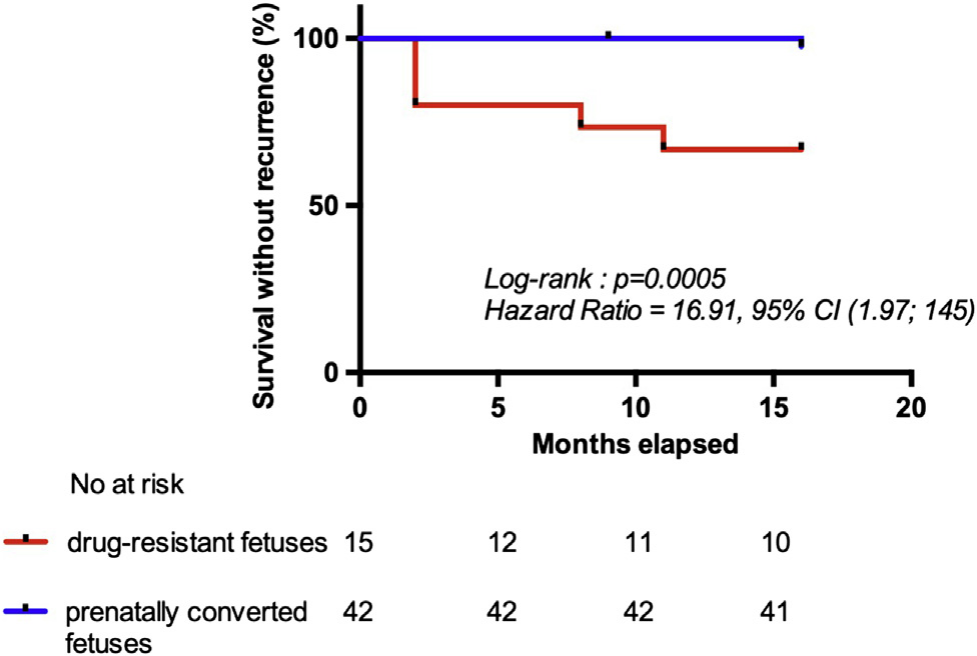
Kaplan-Meier plot of recurrence-free survival beyond the neonatal period (*red line*: drug-resistant fetuses; *blue line*: prenatally converted cases). Follow-up starts at time of discharge.

Antiarrhythmic therapy was given to 24 of 42 (57%) children in group 1: 19 of 42 (45%) prophylactic treatment (as they did not have recurrence during the neonatal period), and 5 of 42 (12%) on maintenance therapy (fetuses presenting neonatal recurrent tachyarrhythmia). All neonates of group 2 were on maintenance antiarrhythmic therapy. Overall, long-term recurrences were observed in 6 of 57 (10.5%) children during the 16 months follow-up. Tachycardia events occurred in 33% (5/15) of children in group 2 vs 2.4% (1/42) of children in group 1 (*P* < .01). The risk of recurrence after hospital discharge and within the 16-month follow-up was significantly higher in the drug-resistant group (*P* < .001, HR = 16.91 [1.97; 145]) (Figure 3).

In the drug-resistant group, all recurrences occurred despite maintenance therapy. AVRT and PJRT were the subtypes at highest risk, with 40% and 60% of them recurring beyond the neonatal period, respectively. Only 1 recurrence was observed in group 1 4 months after the antiarrhythmic treatment was stopped. Within group 1, children who did not recur during the neonatal period also did not recur during follow-up, whether they were on prophylactic treatment or not. No death was observed during the follow-up period.

## Discussion

TPT achieved prenatal conversion to sinus rhythm of fetal tachyarrhythmias in 74% of cases. Prenatal conversion to sinus rhythm was associated with significantly better postnatal outcome. Indeed, the neonatal hemodynamic status was worse in fetuses born arrhythmic and postnatal recurrences of tachyarrhythmias during the first year of life were more frequent. Whereas the most frequent mechanism of tachyarrhythmias in this series was AVRT, rare diagnoses such as PJRT were significantly more frequent in drug-resistant cases, occurring in about 1 of 3 cases.

AVRT is the most common mechanism of fetal and neonatal tachyarrhythmias, accounting for 80%–85% of cases.^2^ Given that a precise diagnosis of the mechanism of the arrhythmia was not achieved in most of the cases prenatally converted to sinus rhythm, and since most of them did not show any postnatal recurrence, we were not able to estimate the overall proportion of AVRT/PJRT in our population of fetal tachyarrhythmia. However, in drug-resistant cases, the proportion of AVRT drops to 38% based on postnatal ECG, with a higher proportion of rarer etiologies such as PJRT and AET in these cases. Indeed, whereas PJRT is found in about 1% of supraventricular tachyarrhythmias in children,^17^^,^^18^ it was common (5/16) in drug-resistant cases in our series. The postnatal management of PJRT is challenging, as it is incessant and resistant to drug therapy. Further, children may present arrhythmic cardiomyopathy in up to 50% of cases,^19^ frequently requiring interventional procedures.^19^^,^^20^ AET and chaotic tachyarrhythmia are also rare diagnoses, but recurrences are rarely observed after 18 months.^21^^,^^22^

Prenatal conversion to sinus rhythm appears to be a major determinant of postnatal evolution and is more likely to occur in the absence of hydrops. Numerous studies have demonstrated that fetal hydrops is an independent predictive factor for treatment failure,^10^^,^^23^ probably because of lower placental transfer and increased fetal distribution volume. This has been well evaluated for digoxin.^24^^,^^25^ However, the rates of prenatal cardioversion remain lower even with other treatment with stable placental transfer, such as flecainide or sotalol.^8^ Thus, pharmacokinetics may not be the only cause for such a failure, which could be partly explained by a more severe disease. This hypothesis is supported by the overrepresentation of AVRT and PJRT in cases of drug-resistant tachyarrhythmia observed in this cohort.

Atrial flutter accounts for 30% of prenatal tachyarrhythmias.^2^ Various studies have evaluated the rate of prenatal conversion to sinus rhythm, with different conclusions. Indeed, Jaeggi and colleagues^10^ found a slower and lower rate of cardioversion compared to other mechanisms, with a 50% rate of cardioversion under transplacental therapy.^10^ More recently, a prospective study showed a higher rate of cardioversion, up to 93% for fetuses presenting flutter without hydrops.^13^ Its management can be challenging, especially when associated with ventricular dysfunction and severe hydrops. In our population, however, cardioversion was achieved in 14 of 17 (82%) of all flutter cases (including hydropic fetuses), which is similar to the overall rate of cardioversion in our population. This rate could be explained by differences in treatment protocol. Indeed, we frequently used 2 or 3 lines of treatment, including amiodarone. Moreover, in a severe hydropic drug-resistant case, in utero transesophageal pacing successfully achieved cardioversion.^15^ This innovative procedure is justified, as recurrences are unlikely once cardioversion is achieved.^2^^,^^26^^,^^27^ However, it may not work in other mechanisms of fetal tachyarrhythmia, for which the risk of recurrence is higher.

Perinatal death occurs, even following prenatal conversion. Indeed, 3 postnatal deaths were observed, and all were hydropic. Although higher in the drug-resistant group (17% vs 6.7%), the difference in perinatal mortality was not found significant. Therefore, these pregnancies should be monitored and delivery in specialized centers appears appropriate, even after prenatal conversion and especially in case of hydrops.

Following the neonatal period, 11% of all children presented long-term recurrences. Once tachyarrhythmia was controlled and sinus rhythm restored during the neonatal period, the rate of long-term recurrences dropped in both groups. Whereas a single recurrence was observed in the prenatally converted group after treatment withdrawal, recurrences were still significantly more frequent in the drug-resistant group and appeared in all cases despite a maintenance therapy, likely because of different underlying mechanisms and particularly with an overrepresentation of rare and challenging pathologies such as PJRT.

In 2017, Hinkle and colleagues^28^ presented a similar evaluation, comparing refractory SVT to nonrefractory SVT. They found refractory fetal SVT to be associated with premature delivery, which is consistent with other observations including our study, but not with postnatal SVT. They concluded that postnatal tachyarrhythmia seems unrelated to the need for prenatal treatment. However, their definition of refractory SVT differs from ours. Indeed, they considered tachyarrhythmia to be refractory when resisting to a single line of treatment. This particular group only represents 67% of our prenatally converted group. The remaining 33% needed 2 or more lines of TPT but were eventually converted during the prenatal period. Moreover, their population differs from ours, as they included fetal intermittent tachyarrhythmia. These differences in population and definitions explain the difference in outcomes.

### Limitations

Although fetal echocardiography is the main tool for the diagnosis of prenatal tachyarrhythmias, as well as for the assessment of cardiac function and of TPT efficacy, it only partly describes the mechanisms of tachyarrhythmias. Atrial flutter is usually easy to diagnose, as atrial activity is regular and has a high rate (usually with a 2:1 conduction), but the distinction between different mechanisms of reentrant tachyarrhythmias remains challenging during the prenatal period. We therefore cannot speculate on prenatal efficacy of different TPT strategies and their association with the underlying mechanism of fetal arrhythmia. In drug-resistant cases, an accurate diagnosis of the electrophysiological mechanism could help in refining TPT and help in predicting postnatal outcomes and planning delivery. New methods to assess fetal cardiac electrophysiology would therefore be useful. Recently, Doshi and colleagues^29^ demonstrated the feasibility of prenatal ECG on 55 women and obtained interpretable results for 50 of them. However, this study did not include cases with tachyarrhythmia, making its ability to accurately characterize tachyarrhythmias speculative. Strand and colleagues^30^ described a new technology for low-cost fetal magnetocardiography, which could enlarge its accessibility. However, application to the management of prenatal tachyarrhythmia has not been performed.

Since all cases of persistent supraventricular tachyarrhythmias were analyzed, including referral for failure of an initial medication, treatment protocol was heterogeneous and could not be evaluated properly. Although sotalol has been shown to be effective, especially in atrial flutter, it was rarely used in this series.^8^^,^^12^ Amiodarone was used in second- or third-line therapy,^31^ despite fetal and maternal side effects on thyroid function.^32^

## Conclusion

The accurate diagnosis of electrophysiological mechanisms is difficult in fetal tachyarrhythmias and remains uncertain during the prenatal period in a significant proportion of cases. Whereas a large majority of cases are easily converted to sinus rhythm with TPT, a significant proportion of fetuses show drug-resistant tachyarrhythmias. In those cases, rare etiologies such as PJRT are more likely, for which the perinatal management is challenging with a high rate of long-term recurrences. Although restoring sinus rhythm significantly reduces postnatal morbidity, adverse perinatal events and recurrences are not rare in the population of fetuses who have been arrhythmic in fetal life, which warrants perinatal and long-term monitoring in specialized centers, even following prenatal successful conversion to sinus rhythm.

## Funding Sources

This research did not receive any specific grant from funding agencies in the public, commercial, or not-for-profit sectors.

## Disclosures

The authors have no conflicts to disclose.

## Authorship

All authors attest they meet the current ICMJE criteria for authorship.

## Patient Consent

All mothers provided their written consent for data use.

## Ethics Statement

Institutional Review Board approval was waived owing to the use of retrospective and de-identified data. The research reported in this paper was conducted according to the principles of the Declaration of Helsinki.
